# Real-time feedback improves chest compression quality in out-of-hospital cardiac arrest: A prospective cohort study

**DOI:** 10.1371/journal.pone.0229431

**Published:** 2020-02-24

**Authors:** Felix Lakomek, Roman-Patrik Lukas, Peter Brinkrolf, Andreas Mennewisch, Nicole Steinsiek, Peter Gutendorf, Hendrik Sudowe, Michael Heller, Robert Kwiecien, Alexander Zarbock, Andreas Bohn

**Affiliations:** 1 Department of Anaesthesiology, Intensive Care and Pain Medicine, Münster University Hospital, Münster, Germany; 2 Department of Anaesthesiology, University Medicine Greifswald, Greifswald, Germany; 3 District of Osnabrück Emergency Medical Services, Osnabrück, Germany; 4 City of Osnabrück Emergency Medical Services, Osnabrück, Germany; 5 GS Elektromedizinische Geräte G. Stemple GmbH, Kaufering, Germany; 6 Institute of Biometrics and Clinical Research (IBKF), Münster University Hospital, Münster, Germany; 7 City of Münster Emergency Medical Services, Münster, Germany; University of Rome 'La Sapienza', ITALY

## Abstract

**Background:**

Current guidelines underline the importance of high-quality chest compression during cardiopulmonary resuscitation (CPR), to improve outcomes. Contrary to this many studies show that chest compression is often carried out poorly in clinical practice, and long interruptions in compression are observed. This prospective cohort study aimed to analyse whether chest compression quality changes when a real-time feedback system is used to provide simultaneous audiovisual feedback on chest compression quality. For this purpose, pauses in compression, compression frequency and compression depth were compared.

**Methods:**

The study included 292 out-of-hospital cardiac arrests in three consecutive study groups: first group, conventional resuscitation (no-sensor CPR); second group, using a feedback sensor to collect compression depth data without real-time feedback (sensor-only CPR); and third group, with real-time feedback on compression quality (sensor-feedback CPR). Pauses and frequency were analysed using compression artefacts on electrocardiography, and compression depth was measured using the feedback sensor. With this data, various parameters were determined in order to be able to compare the chest compression quality between the three consecutive groups.

**Results:**

The compression fraction increased with sensor-only CPR (group 2) in comparison with no-sensor CPR (group 1) (80.1% vs. 87.49%; *P* < 0.001), but there were no further differences belonging compression fraction after activation of sensor-feedback CPR (group 3) (*P* = 1.00). Compression frequency declined over the three study groups, reaching the guideline recommendations (127.81 comp/min vs. 122.96 comp/min, *P* = 0.02 vs. 119.15 comp/min, *P* = 0.008) after activation of sensor-feedback CPR (group 3). Mean compression depth only changed minimally with sensor-feedback (52.49 mm vs. 54.66 mm; *P* = 0.16), but the fraction of compressions with sufficient depth (at least 5 cm) and compressions within the recommended 5–6 cm increased significantly with sensor-feedback CPR (56.90% vs. 71.03%; *P* = 0.003 and 28.74% vs. 43.97%; *P* < 0.001).

**Conclusions:**

The real-time feedback system improved chest compression quality regarding pauses in compression and compression frequency and facilitated compliance with the guideline recommendations. Compression depth did not change significantly after activation of the real-time feedback. Even the sole use of a CPR-feedback-sensor (“sensor-only CPR”) improved performance regarding pauses in compression and compression frequency, a phenomenon known as the ‘Hawthorne effect’. Based on this data real-time feedback systems can be expected to raise the quality level in some parts of chest compression quality.

**Trial registration:**

International Clinical Trials Registry Platform of the World Health Organisation and German Register of Clinical Trials (DRKS00009903), Registered 09 February 2016 (retrospectively registered).

## Background

High-quality chest compression during cardiopulmonary resuscitation (CPR) is one of the most important parameters for a good outcome after cardiac arrest [1]. The current guidelines underline the importance of the quality of chest compression [2]; in particular, minimal interruptions, an appropriate depth and frequency of compressions are highlighted [2]. However, many studies show that chest compression is often carried out poorly in clinical practice, and long interruptions in compression are observed [3, 4, 5].

To improve the quality of chest compression, real-time feedback systems were introduced in order to support emergency medical staff in providing chest compression in conformity with the guidelines. In the current guidelines (2015), real-time feedback systems are mentioned as part of ‘comprehensive CPR quality improvement initiatives’ [2], but due to a lack of data no explicit recommendations are given on the routine use of real-time feedback devices in clinical practice [2].

This present prospective cohort study aimed to analyse whether chest compression quality changes when a real-time feedback system is used in out-of-hospital cardiac arrest.

## Methods

The study was approved by the ethics committees of the regional medical councils (*Ärztekammer Westfalen-Lippe;* (positive vote on 07 December 2015) and *Ärztekammer Niedersachsen;* (positive vote on 12 January 2016)), file no. 2015-604-f-S. The study was also registered with the International Clinical Trials Registry Platform of the World Health Organisation (WHO) and with the German Registry of Clinical Trials (DRKS00009903) (date of application for registration 15 January 2016; date of final registration: 09 February 2016).

Patient’s recruitment of the control group started on 20 January 2016. In this control group the patients received standard of care, so no specific intervention or treatment were performed. All ongoing and related trials for this intervention are registered.

This prospective cohort study consisted of three consecutive study groups. The first study group performed conventional resuscitation (group 1, no-sensor CPR) without use of a feedback sensor. Pauses in compression and compression frequency were analysed on the basis of electrocardiography (ECG) artefacts. In the second group, the feedback sensor was used to measure the compression depth, without offering any real-time feedback (group 2, sensor-only CPR); and in the third study group, the emergency medical staff received audiovisual real-time feedback about the quality of compression (group 3, sensor-feedback CPR). After reaching the case number for each individual group previous calculated in the sample size calculation, the next study phase (group) and thus the next intervention were started. The patients were observed for follow up and short time survival until hospital admission.

The study included all out-of-hospital cardiac arrests occurring in persons who were 18 years of age or older that occurred in the district of Osnabrück (Germany) from January 2016 until March 2018. The physician-based emergency medical service (EMS) in the district of Osnabrück provides services to a population of 508,333.

**Fig 1:**
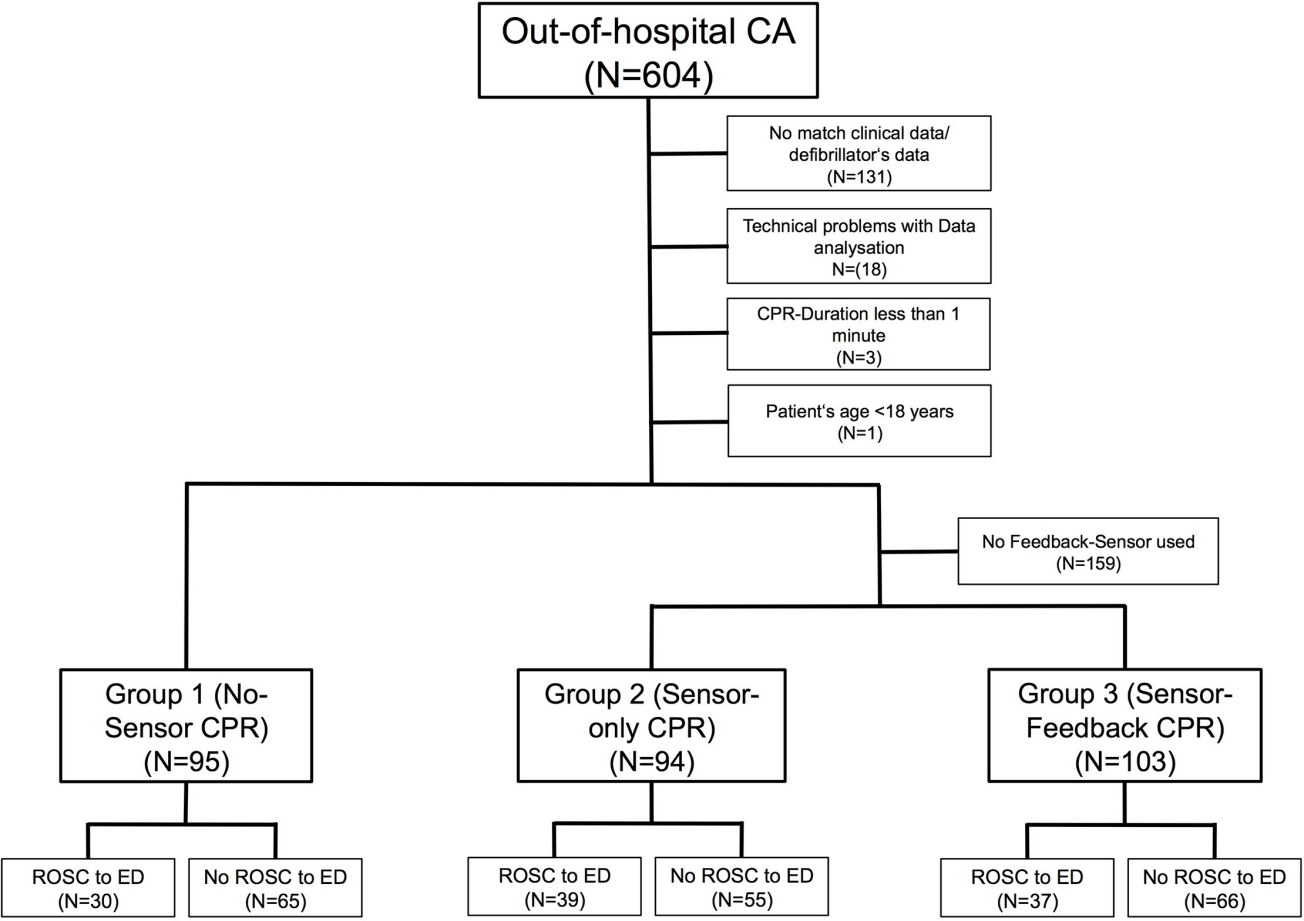
Participants-flowchart.

**Fig 2:**
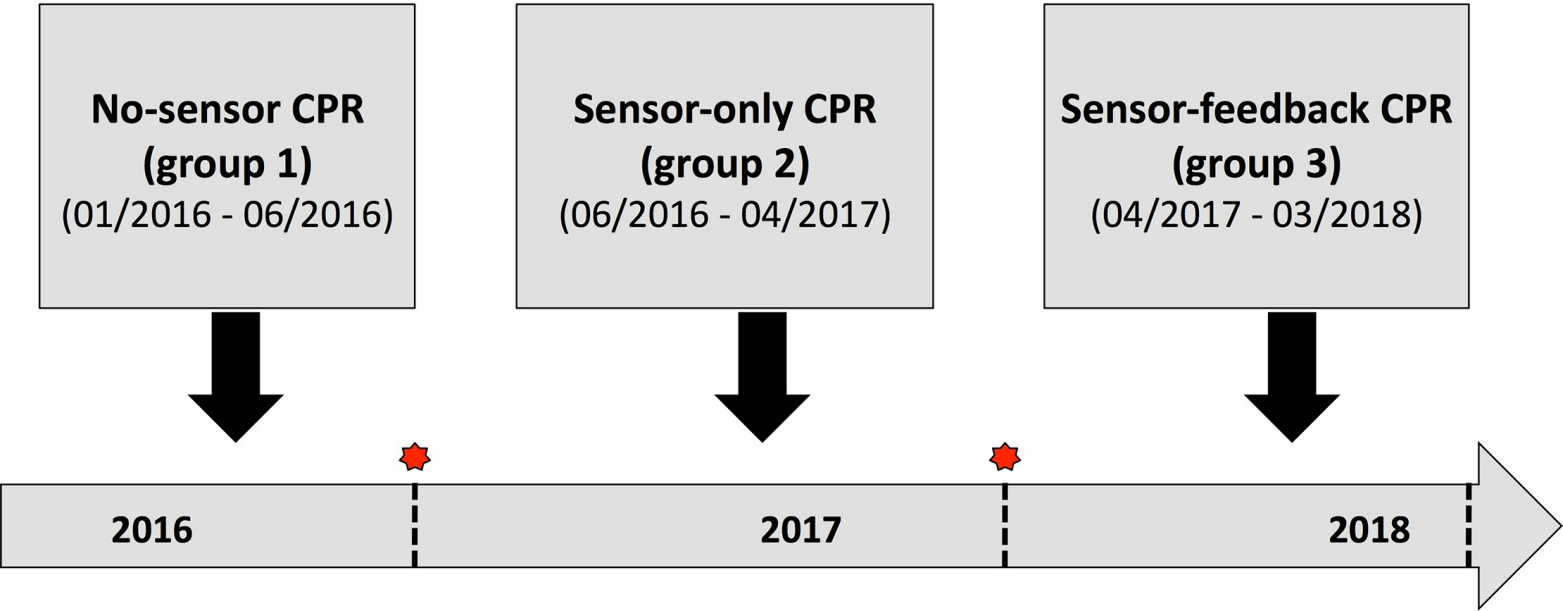
Graphic illustration of the study design.

A total of 604 resuscitations were performed during the study period, and 292 patients were included in the study (group 1: 95; group 2: 94; group 3: 103), so all case numbers calculated in the power analysis could be achieved. 131 cases were excluded, because no clear assignment of clinical data to the defibrillators data was possible. Analysis was not possible in 159 patients due to lack of a feedback sensor, in 18 patients due to technical problems (damaged files), in one case because the patient was under the age of 18, and in three patients because the chest compression time was less than 1 min (Fig 1). The changes between the groups were carried out when the statistical calculated number of cases was reached (Fig 2).

A real-time feedback system incorporated into the corpuls^3®^ defibrillator (with corPatch^®^) was used in this study. The feedback sensor was placed on the patient’s sternum during resuscitation, as described in several studies [3, 6]. The feedback system issues auditory and visual prompts if deviations from the guideline recommendations are detected. In the second group the defibrillator was programmed to measure the compression depth but the feedback function was deactivated in the defibrillator’s software configuration.

Technical instructions were given to the medical staff on how to use the feedback sensor, but no explicit training on resuscitation was provided during the study. Furthermore, several instructions have been attached to the defibrillator to remind the emergency medical staff to use the feedback sensor.

### Data collection

All of the data, such as digital ECG and compression signals recorded by the feedback sensor of the corpuls^3®^ automated external defibrillator, were stored on CF memory cards. These data were matched manually to the patient care reports from the national German resuscitation registry, in which all resuscitations are documented.

### Data analysis

The ECG data collected and the compression signals from the feedback sensor were analysed using the ‘CPRAnalysis’ software program, which was developed in collaboration with GS Elektromedizinische Geräte G. Stemple GmbH, Kaufering, Germany. The program is based on Matlab^®^ R2016b (MathWorks, Inc.) and makes it possible to view resuscitation data as a whole and to annotate landmarks on the ECG. The data were converted into European Data Format (EDF) files using the established corpulsweb.review^®^ (2.0.4 Columbus) program.

Compression artefacts on the ECG were tagged manually by a single investigator (F.L.). The software was able to calculate pauses in compression and compression frequency, as described in many previous studies [3, 7]. Compression artefacts result from changes in thoracic impedance during chest compression [8].

Nonspecific ECG signals were analysed together with a team of experienced emergency physicians (A.B., P.B., R.L.) and were excluded if a clear analysis was not possible.

Every interruption that lasted more than 1.5 s between two compressions was defined as a pause in compression [7]. The longest pause and the chest compression fraction were analysed for each patient. Chest compression fraction is defined as the percentage of time with compression during the cardiac arrest, except for parts with ‘return of spontaneous circulation’ (ROSC).

Defibrillations were tagged automatically. The pre- and post-shock time was calculated as the time interval between the last compression before and the first compression after the defibrillation. ECG segments showing a potentially perfusable heart rhythm were defined as ROSC and therefore excluded from analysis.

Compression frequencies were calculated by the time delta between compressions. Sequenced compression artefacts with no interruptions longer than 1.5 s were grouped together in compression episodes. The mean compression frequency was calculated for every episode. In addition, the percentage of compressions between 100 and 120 compressions/min was detected in order to evaluate the providers’ adherence to the guidelines.

Compression depth was measured using the feedback sensor’s accelerometer. Depth analysis was thus possible only in the sensor-only CPR group and in the sensor-feedback CPR group. The compression bars on the accelerometer were matched to the compression artefacts on the ECG, so that transportation artefacts in the accelerometer signal were identifiable and were excluded from the analysis.

As primary endpoint we defined chest compression quality (chest compression fraction, average compression frequency, average compression depth). Secondary end points were defined as pauses in compression (longest pause, average pre-shock time, longest pre-shock time, average post-shock time, longest post-shock time), compression frequency (maximum episodic frequency, minimum episodic frequency, percentage of compressions 100-120/min) and compression depth (percentage of compressions with a minimum depth of 5 cm, percentage of compressions 5–6 cm in depth). The patients’ short-term survival (any ROSC, ROSC at hospital admission) was defined as a secondary end point as well (Table 1).

**Table 1:** Primary and secondary endpoints.

Primary Endpoint	Secondary Endpoints
Chest compression quality • Chest compression fraction • Average compression frequency • Average compression depth	Short time survival: • Ever ROSC • ROSC at hospital admission Pauses in compression • Longest pause • Average pre-shock time • Longest pre-shock time • Average post-shock time • Longest post-shock time Compression frequency • Maximal episodic frequency • Minimal episodic frequency • Percentage of compressions 100–120 comp./min Compression depth • Percentage of chest compressions with min. 5cm depth • Percentage of chest compressions with 5-6cm depth

The completed datasets were exported into Excel files for further statistical analysis. The patients’ short-term survival was analysed as a secondary end point. For this purpose, clinical data, including ‘any ROSC’ and ‘ROSC at hospital admission’, were taken from the data in the German resuscitation registry.

### Statistical analysis

The sample size calculation and the power analysis were based on the available literature at this time (08/2015), especially on the clinical study “Quality of out-of-hospital cardiopulmonary resuscitation with real time automated feedback: A prospective interventional study (Kramer-Johansen J, et al.)” [3]. This study defines the parameters average compression depth, average compression frequency and chest compression fraction as chest compression quality. Those parameters were therefore chosen for our sample size calculation. The multiple significance level has been set at 5% and for the comparisons with this calculated number of patients for each group a power over 80% was achieved.

Associations between categorical and continuous end points were tested using the Mann–Whitney *U* test and the Kruskal–Wallis test (in this case with estimates plus 95% confidence intervals using the Hodges–Lehmann estimator), and if a corresponding linear model showed normally distributed residuals, a search was made for such associations using the Welch test and ANOVA. Negative binomial regression was used to analyse associations between categorical variables and numerical variables as end points. In addition, associations between two categorical variables were examined using Fisher’s exact test, and associations between categorical end points and time-to-event end points were calculated using the log-rank test and the Kaplan–Meier method.

The following software programs were used for statistical analysis: IBM SPSS Statistics for Windows, version 25.0 (IBM Corporation, Armonk, New York, 2017) and R version 3.1.1 (R Foundation for Statistical Computing, Vienna, Austria, 2014; http://www.R-project.org).

## Results

The Utstein database and demographic characteristics showed comparable results in group 1 (no-sensor CPR), group 2 (sensor-only CPR) and group 3 (sensor-feedback CPR). In the second group (sensor-only CPR), more ventricular fibrillation and pulseless electrical activity were detected as the initial rhythm than in patients with no-sensor CPR and sensor-feedback CPR (groups 1 and 3). Bystander CPR was performed more often in the second and third group (Table 2).

**Table 2:** Utstein characteristics.

Factors	Group 1 (No-sensor CPR)	Group 2 (Sensor-only CPR)	Group 3 (Sensor-feedback CPR)	p-Value (Group1 vs. Group2)	p-Value (Group2 vs. Group3)	p-Value (Group1 vs. Group3)	p-Value (Group1 vs. Group2 vs. Group3)
Resuscitation attempts Status Unwitnessed or unknown Bystander witnessed (%) EMS witnessed (%) Start of CPR Bystander CPR (%) CPR started by EMS (%) Age, mean age (SD), y Gender Woman Men Presenting rhythm Asytole Ventricular fibrillation Pilseless electrical activity or EMD Shock occurred (%) Mean number of compressions (SD)	95 46 (48.42) 44 (46.32) 5 (5.27) 41 (43.16) 53 (55.79) 69.55 (14.18) 31 (32.63) 64 (67.37) 52 (54.74) 22 (23.16) 12 (12.63) 30 (31.58) 1808.14 (1671.83)	94 43 (45.74) 38 (40.43) 13 (13.82) 50 (53.19) 44 (46.81) 69.81 (15.95) 38 (40.43) 56 (59.57) 47 (50.00) 25 (26.60) 20 (21.28) 37 (39.36) 1905.43 (1375.29)	103 53 (51.46) 48 (46.60) 2 (1.94) 55 (53.40) 46 (44.67) 71.02 (12.95) 33 (32.04) 70 (67.96 65 (63.11) 21 (20.39) 14 (13.59) 27 (26.21) 1840.74 (1436.00)	0.170 0.016 0.909 0.292 0.016 0.380	0.019 0.445 0.559 0.237 0.054 0.567	0.656 0.238 0.448 1.000 0.322 0.716	0.070 0.074 0.741 0.395 0.028 0.667

Application of the feedback sensor was delayed in some cases, but there were no statistical differences between sensor delays in the second group (mean 236.57 s) and third group (mean 208.64 s; *P* = 0.35). There were also no statistical differences in the numbers of compressions across all three groups. A total of 540,479 compressions were analysed in the study.

**Fig 3:**
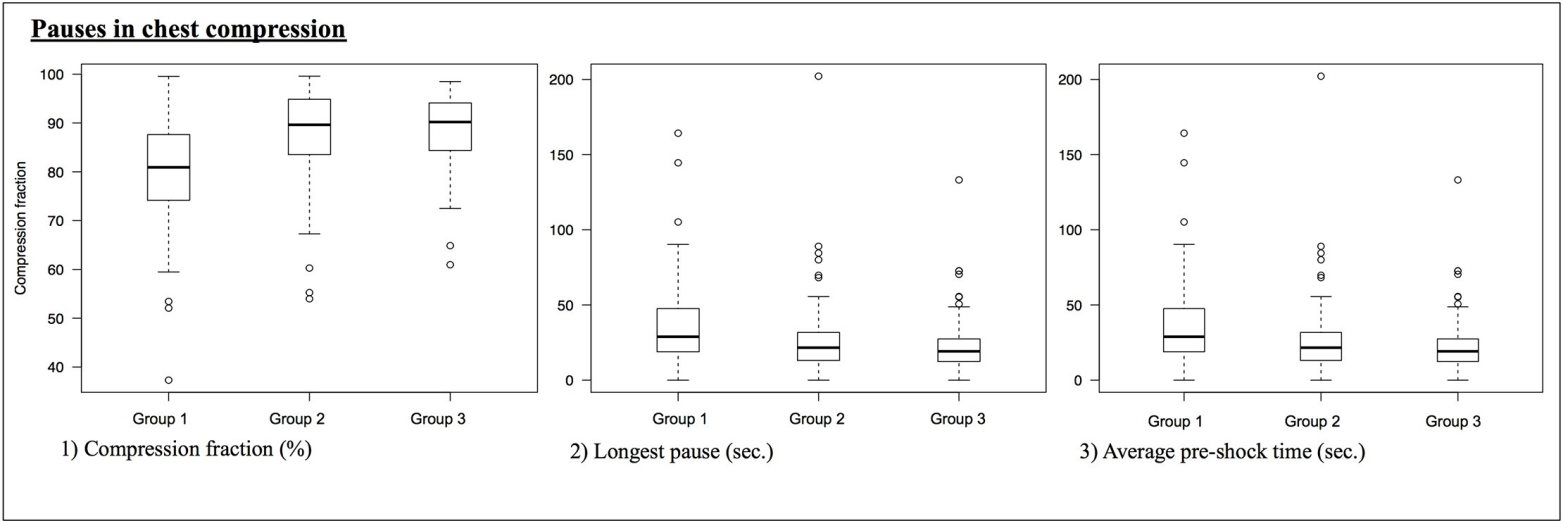
Pauses in chest compression.

Table 2 lists the chest compression data in the three study groups. In comparison with conventional CPR (group 1), the chest compression fraction increased significantly from 80.10% to 87.49% (*P* < 0.001) with sensor-only CPR (group 2). No statistically significant further increase was detected after activation of sensor-feedback CPR (group 3), (group 2 vs. 3: 87.49% vs. 88.85%; *P* = 1.00). The longest pauses per patient decreased from 36.55 s in group 1 to 23.10 s in group 3 (*P* > 0.001) (Fig 3). Peri-shock pauses, average pre-shock time and longest pre-shock time were reduced significantly when the feedback sensor was used. The average post-shock time and longest post-shock time were both reduced after the sensor was introduced (group 1 and 2, Table 3).

**Table 3:** Results in comparison of all study groups on compression quality.

Factors	Group 1 (No-sensor CPR)	Group 2 (Sensor-only CPR)	Group 3 (Sensor-feedback CPR)	p-Value (Group1 vs. Group2)	p-Value (Group2 vs. Group3)	p-Value (Group1 vs. Group3)	p-Value (Group1 vs. Group2 vs. Group3)
Pauses in compression Chest compression fraction % (SD) Longest pause sec. (SD) Average pre-shock time sec. (SD) Longest pre-shock time Average post-shock time sec. (SD) Longest post-shock time sec. (SD) Chest compression frequency Average compression frequency /min (SD) Maximum episodic frequency /min (SD) Minimum episodic frequency /min (SD) Percentage of compressions 100-120/min (SD) Chest compression depth Average compression depth mm (SD) Percentage of compressions with a minimum depth of 5 cm (SD) Percentage of compressions 5–6 cm in depth (SD) Sensor Delay Median sec. ROSC Any ROSC (%) ROSC at hospital admission (%) Running CPR (%)	80.10 (10.21) 36.55 (27.01) 13.86 (12.60) 21.58 (15.83) 5.76 (5.35) 9.75 (8.98) 127.81 (11.58) 144.06 (16.72) 111.87 (13.39) 30.47 (20.79) 43 (45.26) 30 (31.58) 9 (9.47)	87.49 (9.21) 27.43 (25.52) 12.66 (12.88) 21.13 (32.77) 4.46 (9.01) 10.44 (32.41) 122.96 (9.24) 135.89 (12.40) 110.92 (12.21) 35.50 (27.02) 52.49 (11.84) 56.90 (31.74) 28.74 (20.76) 132.77 54 (57.45) 39 (41.49) 11 (11.7)	88.85 (7.15) 23.10 (18.09) 7.69 (7.97) 11.63 (11.02) 3.35 (2.76) 4.83 (4.41) 119.15 (8.86) 130.77 (11.98) 108.07 (11.28) 44.87 (26.19) 54.66 (7.91) 71.03 (28.38) 43.97 (22.95) 99.79 52 (50.49) 37 (35.92) 13 (12.62)	<0.0001 0.001 0.405 0.194 0.049 0.022 0.020 0.0002 0.608 0.154 0.110 0.251	1.000 0.251 0.065 0.110 1.000 0.850 0.008 0.004 0.091 0.014 0.163 0.003 <0.0001 0.347 0.391 0.730	<0.0001 <0.0001 0.008 0.006 0.077 0.012 <0.0001 <0.0001 0.032 <0.0001 0.480 0.575	<0.0001 <0.0001 0.028 0.023 0.096 0.023 <0.0001 <0.0001 0.077 <0.0001 0.241 0.546

**Fig 4:**
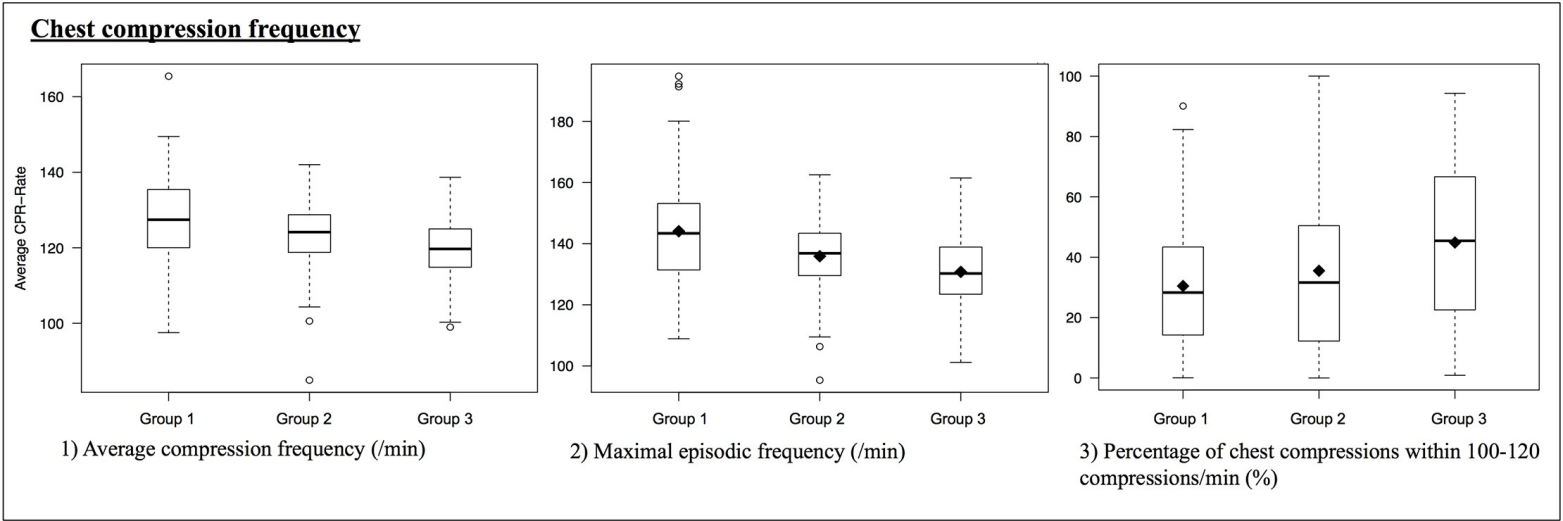
Chest compression frequency.

In comparison with no-sensor CPR (group 1), both sensor-only CPR and activated feedback CPR led to reductions in the average compression frequency (127.81 comp/min in group 1, 122.96 comp/min in group 2; *P* = 0.02; and 119.15 comp/min in group 3; *P* = 0.008). A significant increase in the percentage of compressions between 100 and 120 comp/min was also detected after activation of feedback CPR (from 35.50% with sensor-only CPR to 44.87% with activated sensor-feedback CPR). The maximum episode frequency decreased from 144.06 comp/min through 135.89 comp/min (group 1 vs. group2 2; *P* = 0.0002) to 130.77 comp/min (phase 2 vs. phase 3; *P* = 0.004) (Fig 4).

**Fig 5:**
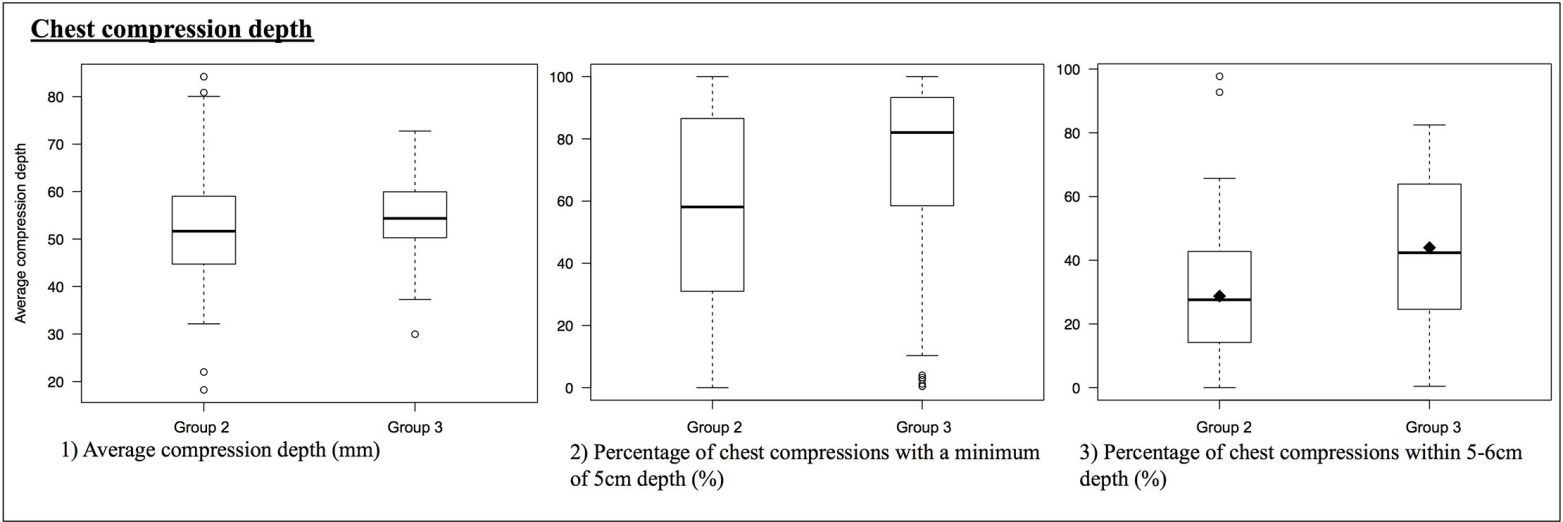
Chest compression depth.

Although introducing the sensor led to an improved chest compression fraction and decreasing chest compression frequency in comparison with no-sensor CPR, the compression depth did not show increase after activation of sensor-feedback CPR (group 2 vs. group 3; 52.49 mm vs. 54.66 mm; *P* = 0.16). With regard to the percentage of chest compressions with a minimum depth of 5 cm, there was an increase from 56.90% in group 2 to 71.03% in group 3 (*P* = 0.003). In addition, the percentage of chest compressions with a depth of 5–6 cm was only 28.74% in group 2 with the sensor-only CPR, and increased to 43.97% (*P* < 0.001) when real-time feedback was used (Fig 5).

No statistically significant differences were noted in short-term survival, including the parameters ‘any ROSC’ and ‘ROSC at hospital admission’ (‘any ROSC’: group 1, 45.26% vs. group 2, 57.45% vs. group 3, 50.49%; *P* = 0.24; ‘ROSC at hospital admission’: group 1, 31.58% vs. group 2, 41.49% vs. group 3, 35.92%; *P* = 0.55). In group 3, more patients reached the hospital with ongoing CPR (group 3, 12.62% vs. group 1, 9.47%).

## Discussion

Providing high-quality chest compressions during CPR—i.e., ensuring limited interruptions in compression and both an appropriate depth and frequency of compressions—continues to be a challenge for paramedical staff. This study investigated the potential of real-time feedback systems for improving the quality of chest compressions in a well-trained professional EMS (Table 3).

In 2005, Wik et al. showed that chest compressions are only applied less than half of the time during a resuscitation event [4] and that most of the compressions applied diverged from the guideline recommendations [4]. However, as Christenson et al. reported, an increased chest compression fraction is an independent predictive factor for better survival [9]. The current guidelines thus underline the importance of ensuring that there are minimal interruptions in compression and that it is performed adequately [2], as this is expected to lead to better hemodynamic perfusion [10] and thus improve the outcome for the patients [11].

### Pauses in compression

In the present study, a chest compression fraction of over 80% was observed in the first study group performing conventional CPR with no Feedback-sensor, showing that raising the issue of the quality of chest compression in recent years has had an effect. Nevertheless, the chest compression fraction achieved by an above-average EMS was significantly improved by the introduction of a feedback sensor. The presence of the sensor alone evidently changed the behaviour of the paramedical staff, reducing interruptions in compression. The compression frequency also showed improved compliance with the guidelines merely through the presence of the feedback sensor. This confounding factor has been described as the ‘Hawthorne effect’—behavioural change due to an awareness of being observed [12]—and has previously been detected in connection with hand hygiene in hospital settings, for example [13]. The compression frequency was further improved when real-time feedback was activated. It can be concluded that the behaviour of emergency medical staff in carrying out CPR partly changed solely due to the fact that they were being observed during the study, and that this still left scope for further improvement.

The study also shows that introducing a real-time feedback system led to a significant decrease in the longest pauses in the cases evaluated. Prolonged pauses have been shown to be associated with decreased survival [14].

Chest compressions have to be interrupted in order to allow interpretation of the ECG and carry out defibrillation. Cheskes et al. described longer peri-shock and pre-shock pauses as being an independent predictor for poor survival in out-of-hospital cardiac arrest [15]. In the present study, both peri-shock pauses and pre-shock pauses were significantly shortened when the feedback sensor was introduced, still leaving potential for further minimisation.

### Compression frequency

Many studies have shown that chest compressions are often poorly performed [4, 16]. The present data show that the compressions applied in the first group with no-sensor CPR were too fast; this has previously been associated with poorer survival [17, 18]. Use of the feedback sensor improved the compression rate, with further improvement when real-time feedback was activated. The mean compression rate reached the guideline recommendation of 100–120/min [2] in the third group when sensor-feedback CPR was activated. The percentage of compressions within the target frequency range also increased.

It is recommended that the chest compression frequency should be kept within 100–120/min in order to restore spontaneous circulation [19, 20]. In the present study, activation of the feedback system effectively reduced high compression rates, while inadequately slow compressions were not observed in any of the groups.

These results show that the configurations of real-time feedback systems, which focus mainly on compression depth, should be modified in order to avoid excessive compression frequencies. Previous studies have reported that there is a noticeable decrease in compression depth when compression rates increase [21]. In an earlier study, our own group showed that compression depth has a significant influence on the patient’s chances of survival [22], is highly accepted by health care providers and various other studies have shown that real-time feedback helps to achieve high-quality CPR [23, 24].

### Compression depth

Our study showed that using a feedback sensor alone, without the feedback being activated, led to an adequate mean compression depth, which did not improve further when the system was activated. Kampmeier et al. previously found that the compression depth did not increase in the majority of cases even when real-time CPR feedback was used to achieve new guideline (2010) recommendation’s compression depth of 5–6 cm [25]. Other authors have concluded that variations in the anatomy of the chest, for example, may lead to incorrect compression depths [26, 27].

However, the proportion of adequately applied compressions increased substantially after activation of real-time feedback during the sensor-feedback CPR phase. More compressions within the explicit guideline recommendation of 5–6 cm were also detected with feedback, confirming the usefulness of real-time feedback in ensuring high-quality chest compressions. These findings are in accordance with those of previous simulator studies that have detected improvements in compression depth [28].

Rittenberger et al. have also shown that the quality of chest compressions declines in proportion to increasing complexity in the resuscitation scenario [29]. Supporting medical staff by providing a CPR feedback system to ensure high-quality chest compression improved the quality of CPR in the present study.

CPR feedback systems should be regarded as a tool for promoting adherence to the guideline and can be successfully used as such [30]. Real-time feedback systems are admittedly only used rarely in clinical practice, but it is recognised that assessing the quality of chest compression is important, even for purposes of comparing resuscitation data [31].

### Limitations

In this study feedback sensor was only used in approximately 50% of cases. The main reason for this was that application of the sensor was omitted in the resuscitation scenario. This can be attributed to the large number of emergency units in the Osnabrück district and the fact that the feedback sensor had to be stored separately from the defibrillation electrodes. When a feedback system is being introduced, intensive motivational work is required to encourage medical staff to accept it. No differences in compliance were noted between the various emergency units, and there were no differences in overall compliance between sensor-only CPR and sensor-feedback CPR. Due to the design of the study it cannot be excluded that chest compression quality improved over time due to the fact that rescuers were aware of being part of a trial on resuscitation quality.

The quality of chest compressions has proven to be outcome-relevant [3, 32]. Despite differences in chest compression quality this study did not show significant differences between the three groups with regard to outcome. This can be attributet to the relatively small number of patients included in the, study which was not designed to show differences in survival. Since many factors play a role in survival of a cardiac arrest (e.g. gender, pre-existing medical condition, underlying cause, witnessed collapse) larger studies are needed to proof the clinical relevance of these findings [33].

## Conclusion

Regarding chest compression frequency, introducing a CPR feedback system improves the quality of chest compressions and helps paramedics comply with the guideline recommendations. The introduction of the feedback sensor alone also helps to reduce pauses in compression and raises the compression fraction with no further improvement after activation of the real-time feedback. Chest compression depth only showed minimal changes when real-time feedback was activated but the ratio of compressions within the range of guideline-recommendations rose. In general the quality of chest compression in the present study was noticeably better than reported in previous studies, and this may be attributable to the emphasis on CPR performance in the current guidelines. Nevertheless, the use of real-time CPR feedback led to further improvements in the compression fraction, frequency and depth of compressions partly caused by the introduction of the feedback sensor alone and partly founded by the activation of real-time feedback. The data show that even EMS that already show good performance can benefit from using a real-time feedback system for further improvement of compression quality parameters. Based on our results we recommend using real-time CPR feedback systems during resuscitations to raise the quality level in chest compression performance especially regarding compression frequency and pauses in compression and to support providers in those time critical situations.

In our study blinded introduction of the sensor alone already led to improved performance, a factor known as the ‘Hawthorne effect’, indicating that improvements of CPR-quality are relatively easy to achieve.

Since there were no statistically significant differences in the short-term survival rates; further studies are needed to investigate the impact of changes in chest compression quality on patient’s survival. Therefore some doubts remain about the economic rationality in introducing such a system into clinical practice.

## Supporting information

S1 Checklist TREND statement checklist. (PDF)

S1 Dataset (CSV)

S2 Dataset (CSV)

S3 Dataset (CSV)

S1 Protocol (DOCX)

S2 Protocol (DOCX)

## Abbreviations

CPR: cardiopulmonary resuscitation
EDF: European Data Format
EMS: emergency medical service
ROSC: return of spontaneous circulation
