# Correction: Real-time feedback improves chest compression quality in out-of-hospital cardiac arrest: A prospective cohort study

**DOI:** 10.1371/journal.pone.0232672

**Published:** 2020-04-28

**Authors:** Felix Lakomek, Roman-Patrik Lukas, Peter Brinkrolf, Andreas Mennewisch, Nicole Steinsiek, Peter Gutendorf, Hendrik Sudowe, Michael Heller, Robert Kwiecien, Alexander Zarbock, Andreas Bohn

There are errors in Tables 2 and 3. Please see the correct tables here.

**Table 2:** Utstein characteristics.

Factors	Group 1 (No-sensor CPR)	Group 2 (Sensor-only CPR)	Group 3 (Sensor-feedback CPR)	p-Value (Group1 vs. Group2)	p-Value (Group2 vs. Group3)	p-Value (Group1 vs. Group3)	p-Value (Group1 vs. Group2 vs. Group3)
Resuscitation attempts	95	94	103				
Status							
Unwitnessed or unknown	46 (48.42)	43 (45.74)	53 (51.46)	0.170	0.019	0.656	0.070
Bystander witnessed (%)	44 (46.32)	38 (40.43)	48 (46.60)				
EMS witnessed (%)	5 (5.27)	13 (13.82)	2 (1.94)				
Start of CPR							
Bystander CPR (%)	41 (43.16)	50 (53.19)	55 (53.40)	0.016	0.445	0.238	0.074
CPR started by EMS (%)	53 (55.79)	44 (46.81)	46 (44.67)				
Age, mean age (SD), y	69.55 (14.18)	69.81 (15.95)	71.02 (12.95)	0.909	0.559	0.448	0.741
Gender							
Woman	31 (32.63)	38 (40.43)	33 (32.04)	0.292	0.237	1.000	0.395
Men	64 (67.37)	56 (59.57)	70 (67.96				
Presenting rhythm							
Asytole	52 (54.74)	47 (50.00)	65 (63.11)	0.016	0.054	0.322	0.028
Ventricular fibrillation	22 (23.16)	25 (26.60)	21 (20.39)				
Pilseless electrical activity or EMD	12 (12.63)	20 (21.28)	14 (13.59)				
Shock occurred (%)	30 (31.58)	37 (39.36)	27 (26.21)				
Mean number of compressions (SD)	1808.14 (1671.83)	1905.43 (1375.29)	1840.74 (1436.00)	0.380	0.567	0.716	0.667

**Table 3:** Results in comparison of all study groups on compression quality.

Factors	Group 1 (No-sensor CPR)	Group 2 (Sensor-only CPR)	Group 3 (Sensor-feedback CPR)	p-Value (Group1 vs. Group2)	p-Value (Group2 vs. Group3)	p-Value (Group1 vs. Group3)	p-Value (Group1 vs. Group2 vs. Group3)
Pauses in compression							
Chest compression fraction % (SD)	80.10 (10.21)	87.49 (9.21)	88.85 (7.15)	<0.0001	1.000	<0.0001	<0.0001
Longest pause sec. (SD)	36.55 (27.01)	27.43 (25.52)	23.10 (18.09)	0.001	0.251	<0.0001	<0.0001
Average pre-shock time sec. (SD)	13.86 (12.60)	12.66 (12.88)	7.69 (7.97)	0.405	0.065	0.008	0.028
Longest pre-shock time	21.58 (15.83)	21.13 (32.77)	11.63 (11.02)	0.194	0.110	0.006	0.023
Average post-shock time sec. (SD)	5.76 (5.35)	4.46 (9.01)	3.35 (2.76)	0.049	1.000	0.077	0.096
Longest post-shock time sec. (SD)	9.75 (8.98)	10.44 (32.41)	4.83 (4.41)	0.022	0.850	0.012	0.023
Chest compression frequency							
Average compression frequency /min (SD)	127.81 (11.58)	122.96 (9.24)	119.15 (8.86)	0.020	0.008	<0.0001	<0.0001
Maximum episodic frequency /min (SD)	144.06 (16.72)	135.89 (12.40)	130.77 (11.98)	0.0002	0.004	<0.0001	<0.0001
Minimum episodic frequency /min (SD)	111.87 (13.39)	110.92 (12.21)	108.07 (11.28)	0.608	0.091	0.032	0.077
Percentage of compressions 100-120/min (SD)	30.47 (20.79)	35.50 (27.02)	44.87 (26.19)	0.154	0.014	<0.0001	<0.0001
Chest compression depth							
Average compression depth mm (SD)		52.49 (11.84)	54.66 (7.91)		0.163		
Percentage of compressions with a minimum depth of 5 cm (SD)		56.90 (31.74)	71.03 (28.38)		0.003		
Percentage of compressions 5–6 cm in depth (SD)		28.74 (20.76)	43.97 (22.95)		<0.0001		
Sensor Delay Median sec.		132.77	99.79		0.347		
ROSC							
Any ROSC (%)	43 (45.26)	54 (57.45)	52 (50.49)	0.110	0.391	0.480	0.241
ROSC at hospital admission (%)	30 (31.58)	39 (41.49)	37 (35.92)	0.251	0.730	0.575	0.546
Running CPR (%)	9 (9.47)	11 (11.7)	13 (12.62)				
