# Supplementary material for: Real-time feedback improves chest compression quality in out-of-hospital cardiac arrest: A prospective cohort study
Source: PLoS One. 2020 Feb 24;15(2):e0229431. doi: 10.1371/journal.pone.0229431 (PMC7039459; doi:10.1371/journal.pone.0229431)
Supplement: S1 Protocol — (DOCX) [file pone.0229431.s005.docx]

**OSCAR – OSNABRÜCK STUDY ON CARDIAC ARREST**

**Studienprotokoll**

Einleitung

Als Einflussfaktor auf das Überleben einer Reanimation wurde insbesondere die Qualität der Thoraxkompression identifiziert (1,2,3,4). Prähospitale wie auch innerklinisch durchgeführte Studien mit professionellem medizinischem Personal zeigen ernüchternde Ergebnisse zur Qualität. Thoraxkompressionstiefe und -frequenz entsprachen nicht den Leitlinienvorgaben (2,4) und häufig wurde die Thoraxkompression unterbrochen, was zu einem Sistieren des Blutflusses führt (5). Auch auf die Leitlinien trainiertes notfallmedizinisches Personal konnte unter Einsatzbedingungen die geforderten Vorgaben zur Reanimationsqualität nicht einhalten (2,4,5). In einer Studie aus Norwegen erreichte kein Rettungsdienst-Team die geforderten Vorgaben der Leitlinien (6). Sowohl aus sicherheitsrelevanten Arbeitsfeldern wie Kraftwerken oder der Luftfahrt (7), als auch aus akutmedizinischen Arbeitsbereichen ist bekannt, dass Stress zu einer Wahrnehmungseinschränkung führen kann. Von Rittenberger wurde dies für den Herz-Kreislauf-Stillstand gezeigt. Mit zunehmender Komplexität verschlechterte sich die Leistungsfähigkeit von Rettungsfachpersonal in simulierten Reanimationsszenarien (8).

Zur Optimierung der Umsetzung einer Reanimation wurden durch die Industrie in den vergangenen Jahren Messinstrumente entwickelt, die die Thoraxkompressionstiefe und –frequenz sowie die Unterbrechung der Kompression während einer Reanimation messen können. Kommt es unter Verwendung dieser Geräte zu Abweichungen vom Standard, so erfolgen audiovisuelle Rückmeldungen an das Personal. Zudem zeichnen diese Geräte Daten aus dem gesamten Verlauf der Reanimation auf und erlauben damit eine Auswertung. Für diese Geräte wurde der Begriff des Echtzeit-Feedback-System (EFS) geprägt (9). Ziel des Einsatzes von Echtzeit-Feedback-Systemen ist die Einhaltung der Leitlinienvorgaben zu Thoraxkompressionstiefe und –frequenz sowie die Minimierung von Unterbrechungen der Kompression. Die aktuellen, im Jahr 2010 publizierten Leitlinien zu Reanimation betonen die Wichtigkeit der Sicherstellung einer „qualitativ hochwertigen Reanimation“ als einen der wichtigsten Prädiktoren für das Patientenüberleben (10,11). Die Leitlinien geben für die Reanimation von Erwachsenen eine Thoraxkompressionstiefe von 5 - 6 cm vor, gefolgt von einer Entlastung des Thorax.

Grund für diese Forderung zur Thoraxkompressionstiefe ist der in der Zwischenzeit belegte Zusammenhang zwischen Thoraxkompressiontiefe und Reanimationserfolg (4,12). Eine Frequenz von 100 - 120 Thoraxkompressionen pro Minute soll eingehalten werden (10,11). Da jede Unterbrechung der Thoraxkompression zu einem Abfall des koronaren und zerebralen Perfusionsdruckes führt und die Erfolgschancen einer Reanimation sinken lässt (3,13,14) sollen Unterbrechungen, z.B. zur EKG-Interpretation, auf ein Mindestmaß beschränkt bleiben und nur am Ende eines sog. Reanimationszyklus (10) erfolgen. Die Dauer eines Zyklus wird auf zwei Minuten festgelegt.

Ziel/Hypothese:

Verglichen mit einer konventionellen Reanimation verändert ein Echtzeit-Feedbacksystem mit Erfassung der Thoraxkompression (TK) die Qualität der Reanimation im außerklinischen Herzstillstand hinsichtlich Kompressionstiefe, -frequenz und Unterbrechung der Kompression.

Endpunkt:

1.Primärer Endpunkt:

1.1. Veränderung der Qualität der Reanimation (Kompressionstiefe, -frequenz und Unterbrechung der Kompression).

1.2. Erreichen der durch die Leitlinien des European Resuscitation Council (ERC) vorgegebenen Ziel-Parameter hinsichtlich Kompressionstiefe, -frequenz und Unterbrechung der Kompression.

2.Sekundärer Endpunkt:

2.1. Veränderung des Kurzzeit-Überlebens der reanimierten Patienten definiert als Erreichen des Krankenhauses mit eigenem Kreislauf (Return of spontaneous circulation, ROSC).

Methode

Ad 1.a Kompressionstiefe:

Das Echtzeit-Feedbacksystem (EFS) ist in einen Defibrillator integriert (Fa. corpuls, Kaufering, Deutschland ) und beinhaltetet eine Sternal-Elektrode mit Accelerometer (CorPatch CPR; corpuls, Kaufering, Deutschland). Der Sternalsensor wird auf dem Druckpunkt in der Mitte des Sternums platziert. Er besitzt einen Piezo-Kristall, der durch die Thoraxkompression in sagittale Bewegung gebracht wird. Das EFS erfasst Geschwindigkeit und Zeit jeder einzelnen Thoraxkompression. Aus diesen beiden Variablen errechnet es in Echtzeit Thoraxkompressionstiefe. Thoraxkompressionen werden mit einer Genauigkeit von ± 6,25 mm erfasst (15,16).

Ad 1.b.Kompressionsfrequenz:

Die Kompressionsfrequenz wird über EKG-Artefakte aus dem Monitor-EKG nachträglich abgeleitet. Hierzu wird die Ableitung II, mittels der selbstklebenden Defibrillationselektroden fortwährend gemessen und auf einer Speicherkarte (CF-Karte) abgespeichert. Dieses EKG kann unter Zuhilfenahme der Software corView2 angezeigt und analysiert werden. Thoraxkompressionen lassen sich auf dem EKG von anderen elektrischen Aktionen des Herzens unterscheiden. Die Kompressionsfrequenz wird analog ermittelt.


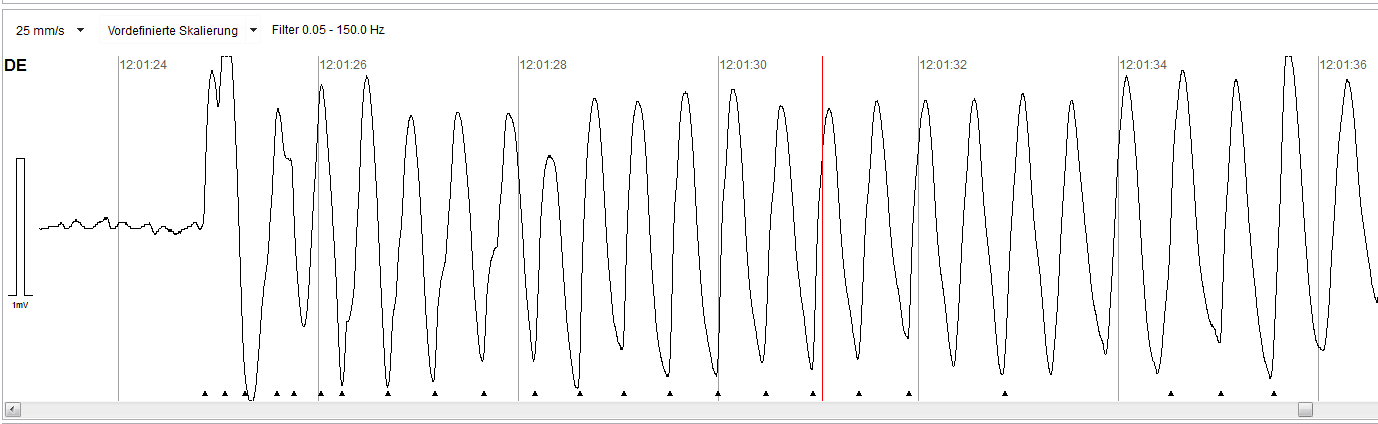


**EKG Intervall mit Thoraxkompression (Ansicht in corView2)**


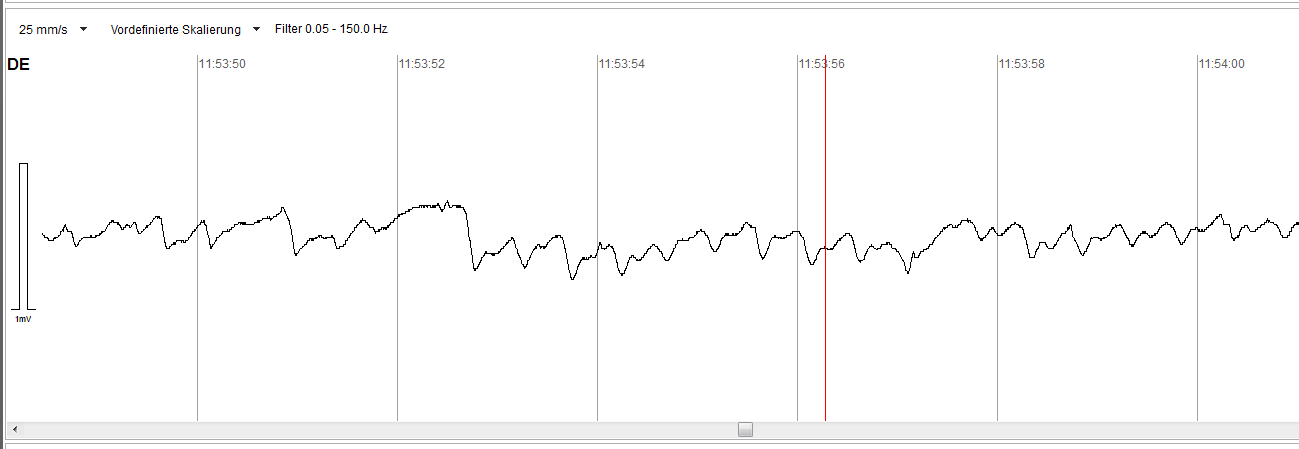


**EKG Intervall ohne Thoraxkompressionen (Ansicht in corView2)**

Ad 1.c.Unterbrechungen der Kompression:

Die Unterbrechungen der Kompression („Hands-off“) werden über Artefakte aus dem EKG nachträglich abgeleitet.

- Summe Pausen:

Alle ermittelten Hands-off Zeiträume zwischen der ersten und der letzten Druckmassage. Ein Zeitraum ohne Druckmassage ist mit einem Schwellenwert von mind. 1,5 sek definiert (Hands-off Zeiträume sind dabei Zeiträume in denen keine Thoraxkompressionen stattfinden)

- Längste Pause:

längste Hands-off Pause in Sekunden

- Anzahl Pausen >10s:

Anzahl aller Hands-off Pausen größer als 10s

- Mittleres Zeitintervall bis zur Defibrillation („Pre-Shock Pause“):

Durchschnittszeit in Sekunden aller Pre-Shock . Wird nur für Hands-Off Zeiträume mit einem gelieferten Schock-Ereignis berechnet. Bei mehreren Schocks innerhalb eines Hands-off Zeitraums gilt immer die erste Defibrillation.

- Post-Shock Pause:

Durchschnittszeit in Sekunden aller Post-Schock Pausen. Wird nur für Hands-Off Zeiträume mit einem gelieferten Schock-Ereignis berechnet. Bei mehreren Schocks innerhalb eines Hands-off Zeitraums gilt immer die erste Defibrillation.

- Peri-Shock Pause:

Durchschnittszeit aller Hands-Off Zeiträume mit einem oder mehreren Defibrillationen.

- Kompressionsanteil (chest compression fraction):

Verhältnis von von Hands-On-Zeit zu Hands-Off-Zeit (also das Verhältnis der Zeiten in den Thoraxkompression durchgeführt werden im Verhältnis zu den Zeiten in denen keine Thoraxkompressionen durchgeführt werden.

Ad 2. Kurzzeit-Überleben:

Die Reanimationsergebnisse (Kurzzeitüberleben) werden im Deutschen Reanimationsregister® erfasst und ausgewertet.

Messgrößen:

- Endpunkt 1.1: Erfassung einer statisch signifikanten Veränderung der genannten Qualitäten (Thoraxkompressionsfrequenz und Unterbrechung der Kompression) zur Phase 1 in den Phasen 2 und 3. Erfassung einer Veränderung der Thoraxkompressionstiefe in den Phasen 2 und 3. Als Unterbrechungen werden Pausen länger als 1,5 s gezählt, bei denen gleichzeitig das EKG keinen Hinweis auf einen perfundierenden Herzrhythmus i.S.e. Spontankreislaufes gibt (18).
- Endpunkt 1.2 Prozentuale Abweichung von den ERC-Leitlinien hinsichtlich Kompressions-Tiefe: 5.0-6,0 cm (19), Kompressions-Frequenz: 100/min (19)
- Endpunkt 2: Erreichen einer Krankenhausaufnahme mit spontanem Kreislauf (ROSC) im Reanimationsregister dokumentiert.

Einschlusskriterien:

- Alle Patienten mit einem nicht-traumatischen Herz-Kreislauf-Stillstand, bei denen ein Reanimationsversuch unternommen wurde, ab dem 18. Lebensjahr. Ausgeschlossen sind Häftlinge.
- Reanimationen inkl. Thoraxkompression mit einer Dauer von mehr als einer Minute.

Ablauf:

3-Phasen-Ablauf:

In der Ersten Phase werden 95 Patientendaten eingeschlossen, in Phase 2 und 3 jeweils 92 (insgesamt 279). Diese Größenordnung entstammt dem biometrischen Gutachten (s. Anlage 1). Die kreisfreie Stadt Osnabrück und der Landkreis Osnabrück haben zusammengenommen eine Einwohnerzahl von 506 000 Einwohnern (Stand Dez. 2013) (17). Es ist jährlich mit ungefähr 300-400 außerklinischen Reanimationsversuchen zu rechnen.

- **Phase 1:** Datensätze werden ohne Anwendung des Accelerometers (Echtzeit-Feedbacksystem) gewonnen. Die Analyse von Thoraxkompressionsfrequenz und Unterbrechungen erfolgt über die EKG-Auswertung. Die Daten des Reanimationsregisters werden zur Analyse der klinischen Verläufe genutzt.
- **Phase 2:** Datensätze werden unter Verwendung des Accelerometers gewonnen, ohne dass das Reanimationsteam ein Echtzeit-Feedback bekommt. Die Analyse von Thoraxkompressionsfrequenz und Unterbrechungen erfolgt über die EKG-Auswertung. Die Daten des Reanimationsregisters werden zur Analyse der klinischen Verläufe genutzt.
- **Phase 3**: Datensätze werden unter Verwendung des Accelerometers gewonnen, das Reanimationsteam bekommt ein Echtzeit-Feedback zu Qualität. Die Analyse von Thoraxkompressionsfrequenz und Unterbrechungen erfolgt über die EKG-Auswertung. Die Daten des Reanimationsregisters werden zur Analyse der klinischen Verläufe genutzt.

Datenfluss der Gerätedaten:

1. Die Patientendaten der jeweiligen Reanimation werden über mobile Datenübertragung auf einen Server geladen und von dort zur Auswertung abgerufen. Dies entspricht dem üblichen Verfahren in den Rettungsdiensten. Die Reanimationseinsätze werden nachträglich im Kollektiv der Einsätze identifiziert.
2. Die Daten zur Reanimationsqualität werden über mobile Datenübertragung auf einen Server geladen und von dort nach Identifikation der Einsätze (s. Punkt 1) zur Auswertung abgerufen. Die Datensätze werden anonymisiert an die Firma corpuls übersendet:


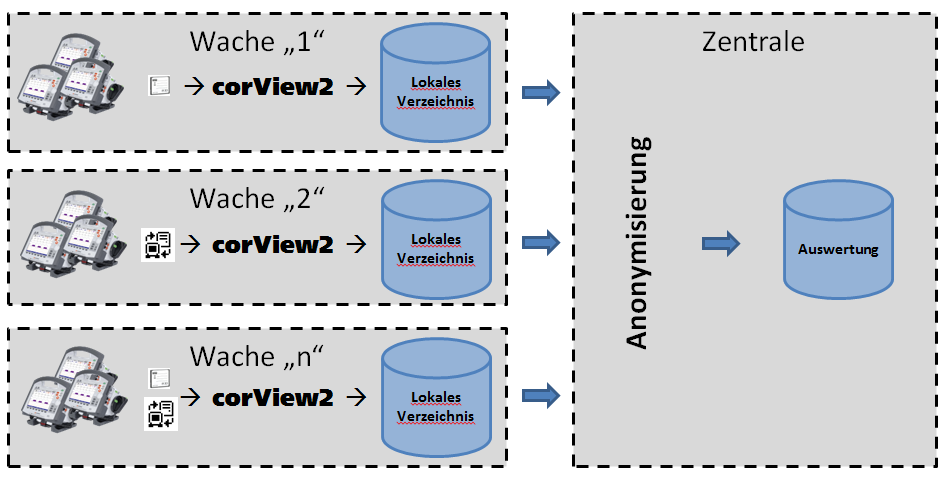


Die Anonymisierung wird über Filter erfolgen.Die einzelnen Daten sind im Folgenden aufgeführt. Alle Daten, die direkt oder in der Kombination mit anderen Daten zur Identifikation eines Patienten führen könnten werden entfernt. Die Inhalte sind in der Auswertungsdatenbank nicht mehr vorhanden und können auch nicht mehr nachträglich wiederhergestellt werden.

| Patientendaten | | | | |
| --- | --- | --- | --- | --- |
| **Art der Daten** | identifizierend?  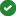 = ja 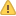 = evt. resp. in Kombination 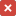 = nein | PHI?  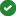 = ja 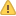 = evt. 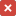 = nein | Maßnahmen | Ergebnis  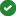 = k.P. 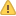 = Achtung 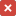 = Anw. BDSG |
| **Patienten-ID** | 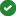 | 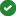 | Daten werden entfernt. | 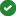 |
| **Fallnummer** | 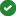 | 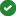 | Daten werden entfernt. | 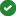 |
| **Vorname** | 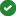 | 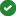 | Daten werden entfernt. | 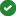 |
| **Nachname** | 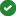 | 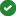 | Daten werden entfernt. | 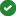 |
| **Geschlecht** | 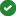 | 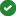 | Daten werden entfernt. | 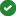 |
| **Geburtsdatum** | 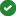 | 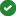 | Daten werden entfernt. | 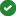 |
| **Alter** | 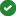 | 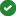 | Daten werden entfernt. | 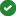 |
| **Gewicht** | 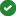 | 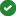 | Daten werden entfernt. | 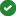 |
| **Größe** | 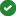 | 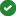 | Daten werden nicht erfasst | 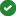 |
| **Adresse** | 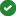 | 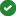 | Daten werden entfernt. | 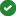 |
| **Ort** | 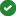 | 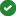 | Daten werden entfernt. | 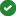 |
| **Versicherten-Nr** | 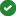 | 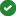 | Daten werden entfernt. | 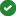 |
| **KK/Kostenträger** | 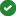 | 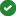 | Daten werden entfernt. | 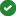 |
| **Versicherten Karten-Nr** | 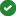 | 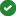 | Daten werden entfernt. | 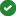 |
| **Transportmittel** | 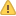 | 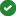 | Daten werden entfernt. | 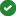 |
| **Funkkennung** | 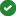 | 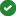 | Daten werden entfernt. | 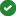 |
| **Geräte-ID** | 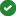  in Kombination mit Organisation |  | Daten werden entfernt. | 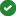 |
| **Zeitpunkt des Gerätestarts (Einsatzstart)** | 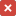 | 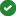 | identifizierende Daten entfernen, damit sind auch diese Daten kein PHI mehr | 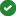 |
| **Dauer des Einsatzes (Einsatzende)** | 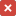 | 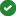 | identifizierende Daten entfernen, damit sind auch diese Daten kein PHI mehr | 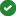 |
| **Seriennummer der einzelnen Gerätemodule** | 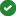 |  | Daten werden entfernt. | 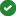 |
| **Seriennummer der einzelnen Geräteoptionen** | 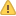 |  | Daten werden entfernt. | 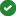 |
| **Kontinuierlich aufgezeichnete Kurven** | 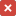 | 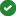 | identifizierende Daten entfernen, damit sind auch diese Daten kein PHI mehr | 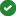 |
| **Kontinuierlich aufgezeichnete Vitalparameter** | 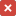 | 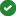 | identifizierende Daten entfernen, damit sind auch diese Daten kein PHI mehr | 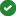 |
| **12-Kanal-EKG** | 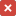 | 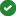 | identifizierende Daten entfernen, damit sind auch diese Daten kein PHI mehr | 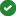 |
| **12-Kanal-EKG Vermessung, Diagnose und Therapieempfehlung (HES)** | 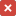 | 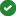 | identifizierende Daten entfernen, damit sind auch diese Daten kein PHI mehr | 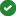 |

Datenfluss der Patientendaten:

Der Einsatz wird im Deutschen Reanimationsregister dokumentiert und von dort zur Auswertung abgerufen. Im Reanimationsregister wird der Einsatz pseudonymisiert unter einer Einsatznummer gespeichert und kann von dort zur Auswertung über die Einsatznummer abgerufen werden, ohne das auf Daten, die eine direkte Identifikation des Patienten ermöglichen, zugegriffen werden kann (s. Anlage 2).

Zusammenführung von Patienten- und Gerätedaten:

Die Zusammenführung erfolgt auf einem Rechner des Universitätsklinikums Münster wie dargestellt. Als Bezugsgröße der Patientendaten gilt hierbei die sogenannte Einsatznummer.

Statistik:

Die statistischen Berechnungen wurden auf Basis des Wissens aus der Studie Kramer- Johansen et al. „Quality of out-of-hospital cardiopulmonary resuscitation with real time automated feedback: a prospective interventional study” ermittelt.

1. Zu Endpunkt 1.1: Die Daten der Phase 1 werden hinsichtlich Thorax-kompressionsfrequenz und Unterbrechung als Referenz verwendet; die Daten der Phase 2 werden als Referenz für die Kompressionstiefe genutzt. Diese Phasen werden mit den darauffolgenden Phasen hinsichtlich der Qualität verglichen.
2. Zu Endpunkt 1.2: Die Daten werden phasenspezifisch hinsichtlich der prozentualen Abweichung von den ERC-Reanimations-Leitlinien beurteilt.
3. Es wird geprüft ob phasenspezifische Unterschiede im Kurzzeitüberleben erkennbar sind.

Kompressionsfrequenz

Unterbrechung der Kompression

Kompressionstiefe

Phase 2

Phase 1

Phase 3

Phase 2

Phase 3

Das multiple Signifikanzniveau ist bei 5% angesetzt, und die Power soll stets bei ≥ 80% liegen (Fehler 2. Art ≤ 20%).

Es gibt für jede Zielgröße einen oder mehrere Vergleiche. Das multiple Signifikanzniveau von 5% wird auf die Zielgrößen nach Bonferronie in drei Teile (gewichtet) aufgeteilt:

1. Für die Vergleiche bzgl. Komressionstiefe wird ein lokales Signifikanzniveau von 1/4 * 5% aufgewendet. Hier werden nur die Gruppen Phase 2 und Phase 3 miteinander bzgl. des Mann-Whitney-U-Tests zum lokalen Signifikanzniveau 1/4 * 5% miteinander verglichen.
2. Für die Vergleiche bzgl. Kompressionsfrequenz wird ein lokales Signifikanzniveau von 1/4 * 5% aufgewendet. Hier werden die Gruppen Phase1, Phase 2 und Phase 3 miteinander paarweise zum lokalen Signifikanzniveau 1/4 * 5% miteinander verglichen. Das ergibt 3 Gruppenvergleiche. Das multiple Testproblem wird hier mittels der Verwendung des Abschlusstest gelöst (für die Paarvergleiche wird der Mann-Whitney-U-test verwendet, für die 3 Gruppen-Vergleiche (Schnitthypothese) wird der Kruskal-Wallis-Test verwendet.).
3. Für die Vergleiche bzgl. Kompressionspause wird ein lokales Signifikanzniveau von 1/2 * 5% aufgewendet. Hier werden die Gruppen Phase1, Phase 2 und Phase 3 miteinander paarweise zum lokalen Signifikanzniveau 1/2 * 5% miteinander verglichen. Das ergibt 3 Gruppenvergleiche. Das multiple Testproblem wird hier mittels der Verwendung des Abschlusstest gelöst (für die Paarvergleiche wird der Mann-Whitney-U-test verwendet, für die 3 Gruppen-Vergleiche (Schnitthypothese) wird der Kruskal-Wallis-Test verwendet.).

Powerkalkulation und Fallzahlen:

Für die Behandlungsgruppe Phase 1 werden 95 Patienten benötigt, für die Behandlungsgruppe Phase 2 werden 92 Patienten benötigt, und Für die Behandlungsgruppe Phase 3 werden 92 Patienten benötigt. Insgesamt werden für dieses Projekt also 95 + 92 + 92 = 279 Patienten benötigt.

1. Für die Vergleiche bzgl. Komressionstiefe wird mit diesen Patientenzahlen eine Power von 82.1% erreicht.
2. Für die Vergleiche bzgl. Kompressionsfrequenz wird mit diesen Patientenzahlen eine Power von 86.5% erreicht (= Wahrscheinlichkeit, dass alle drei Paarvergleiche ein signifikantes Resultat ergeben).
3. Für die Vergleiche bzgl. Kompressionspause wird mit diesen Patientenzahlen eine Power von 80.1% erreicht (= Wahrscheinlichkeit, dass alle drei Paarvergleiche ein signifikantes Resultat ergeben).

Weitere geplante statistische Analysen: Darüber hinaus werden quantitative Schätzungen (Hodschges-Lehmann-Schätzer) zzgl. 95% Konfidenzintervalle für die Gruppenunterschiede berechnet, und als explorative Fragestellung werden alle Vergleiche zusätzlich nach Geschlecht getrennt umgesetzt.

Literaturverzeichnis:

(1) Abella BS, Alvarado JP, Myklebust H, Edelson DP, Barry A, O'Hearn N, et al. (2005) Quality of cardiopulmonary resuscitation during in-hospital cardiac arrest. JAMA 293 (3): 305-310

(2) Abella BS, Sandbo N, Vassilatos P, Alvarado JP, O'Hearn N, Wigder HN, et al. (2005) Chest compression rates during cardiopulmonary resuscitation are suboptimal: a prospective study during in-hospital cardiac arrest. Circulation 111 (4): 428-434

(3) Christenson J, Andrusiek D, Everson-Stewart S, Kudenchuk P, Hostler D, Powell J, et al. (2009) Chest compression fraction determines survival in patients with out-of-hospital ventricular fibrillation. Circulation 120 (13): 1241-1247

(4) Kramer-Johansen J, Myklebust H, Wik L, Fellows B, Svensson L, Sorebo H, et al. (2006) Quality of out-of-hospital cardiopulmonary resuscitation with real time automated feedback: a prospective interventional study. Resuscitation 71 (3): 283-292

(5) Wik L, Kramer-Johansen J, Myklebust H, Sorebo H, Svensson L, Fellows B, et al. (2005) Quality of cardiopulmonary resuscitation during out-of-hospital cardiac arrest. JAMA 293 (3): 299-304

(6) Bjorshol CA, Myklebust H, Nilsen KL, Hoff T, Bjorkli C, Illguth E, et al. (2010) Effect of socioemotional stress on the quality of cardiopulmonary resuscitation during advanced life support in a randomized manikin study. Crit.Care Med.

(7) Goh J WD (2002) Aviat Space Environ Med 73 (73): 817–22

(8) Rittenberger JC, Guimond G, Platt TE, Hostler D (2006) Quality of BLS decreases with increasing resuscitation complexity. Resuscitation 68 (3): 365-369

(9) Lukas RP, Van Aken H, Engel P, Bohn A (2011) Real-time feedback systems for improvement of resuscitation quality. Anaesthesist

(10) Deakin CD, Nolan JP, Soar J, Sunde K, Koster RW, Smith GB, et al. (2010) European Resuscitation Council Guidelines for Resuscitation 2010 Section 4. Adult advanced life support. Resuscitation 81 (10): 1305-1352

(11) Koster RW, Baubin MA, Bossaert LL, Caballero A, Cassan P, Castren M, et al. (2010) European Resuscitation Council Guidelines for Resuscitation 2010 Section 2. Adult basic life support and use of automated external defibrillators. Resuscitation 81 (10): 1277-1292

(12) Edelson DP, Abella BS, Kramer-Johansen J, Wik L, Myklebust H, Barry AM, et al. (2006) Effects of compression depth and pre-shock pauses predict defibrillation failure during cardiac arrest. Resuscitation 71 (2): 137-145

(13) Berg RA, Sanders AB, Kern KB, Hilwig RW, Heidenreich JW, Porter ME, et al. (2001) Adverse hemodynamic effects of interrupting chest compressions for rescue breathing during cardiopulmonary resuscitation for ventricular fibrillation cardiac arrest. Circulation 104 (20): 2465-2470

(14) Eftestol T, Sunde K, Steen PA (2002) Effects of interrupting precordial compressions on the calculated probability of defibrillation success during out-of-hospital cardiac arrest. Circulation 105 (19): 2270-2273

(15) Bohn A, Van Aken H, Weber TP, Weber B, Lukas R (2012) Effects and limitations of an AED with audiovisual feedback for cardiopulmonary resuscitation. Resuscitation 83 (1): e9

(16) Zoll Medical Corporation [Erfinder]; Anonymous [Patentanmelder] (2011) Zoll E-Series (R) Benutzerhandbuch.

(17) [Landesbetrieb für Statistik und Kommunikationstechnologie Niedersachsen, 102 Bevölkerung – Basis Zensus 2011, Stand 31. Dezember 2013 (Tabelle K1020014)](http://www1.nls.niedersachsen.de/statistik/)

(18) Kramer-Johansen, J.; Edelson, D.P.; Losert, H.; et al Uniform reporting of measured quality of cardiopulmonary resuscitation. Resuscitation 2007; 412

(19) ERC Guidelines for Resuscitation; www.erc.edu

Anlagen:

- Anlage 1: Biometrisches Gutachten
- Anlage 2: Ethikvotum des Deutschen Reanimationsregisters
