# Supplementary material for: Real-time feedback improves chest compression quality in out-of-hospital cardiac arrest: A prospective cohort study
Source: PLoS One. 2020 Feb 24;15(2):e0229431. doi: 10.1371/journal.pone.0229431 (PMC7039459; doi:10.1371/journal.pone.0229431)
Supplement: S2 Protocol — (DOCX) [file pone.0229431.s006.docx]

**OSCAR – OSNABRÜCK STUDY ON CARDIAC ARREST**

**Study protocol**

Introduction

In particular, the quality of chest compression quality during resuscitation was identified as an influencing factor for survival of cardiac arrest (1,2,3,4). Pre-hospital as well as in-hospital studies with professional medical staff show sobering quality results. Thorax compression depth and frequency did often not meet the guidelines (2.4) and chest compression was frequently discontinued resulting in blood flow arrest (5). Even emergency medical personnel trained on the guidelines were unable to comply with the required recommendations for resuscitation quality in resuscitation scenario (2,4,5). In a study from Norway, no rescue team reached the required guidelines recommendations on resuscitation quality (6). Both from safety-relevant fields of work such as power plants or aviation (7), as well as from acute medical work areas is known that stress can lead to a limitation of perception. Von Rittenberger were able to show this for behavior in cardiac arrest situations. With increasing complexity, the performance of Emergency medical staff deteriorated in simulated resuscitation scenarios (8).

In order to optimize the chest compression quality, the industry has developed in recent years measuring instruments that can measure the chest compression depth and frequency as well as the interruption of compression during resuscitation. If deviations from the standard occur when using these devices, audiovisual feedback is given to the staff. In addition, these devices record data from the entire course of the resuscitation and thus allow an evaluation. These devices have been named real-time feedback system (RTF)(9). The aim of using real-time feedback systems is to comply with the guidelines for chest compression depth and frequency and to minimize interruptions in compression. The current reanimation guidelines published in 2010 emphasize the importance of ensuring "high-quality resuscitation" as one of the most important predictors of patient survival (10,11). The guidelines provide a chest compression depth of 5 - 6 cm for adult resuscitation, followed by total chest relief.

The reason for this demand for thorax compression depth is the correlation between thoracic compression depth and resuscitation success, which has been documented in different studies (4,12). Furthermore a frequency of 100 - 120 chest compressions per minute should be maintained (10,11). Any disruption of chest compression results in a decrease in coronary and cerebral perfusion pressure and decreases the chances of survival (3,13,14), interruptions, for example for ECG interpretation, remain restricted to a minimum and are only advised at the end of a resuscitation cycle (10). The duration of a cycle is set to two minutes.

Aim/Hypothesis:

Compared with conventional resuscitation, a real-time feedback system changes the quality of resuscitation in out-of-hospital cardiac arrest when considering compression depth, rate, and pauses in compression.

Endpoint:

1.Primary Endpoint:

1.1. Change in the quality of resuscitation (compression depth, frequency and pauses in compression).

1.2. Reaching the recommended target parameters set by the guidelines of the European Resuscitation Council (ERC) for compression depth, frequency and pauses in compression.

2.Secondary Endpoint:

2.1. Change in short-term survival of resuscitated patients defined as reaching the hospital with return of own circulation (ROSC).

Methods

Ad 1.a compression depth:

The real-time feedback system (RTF) is integrated into a defibrillator (corpuls, Kaufering, Germany) and includes a sternal electrode with accelerometer (CorPatch CPR, corpuls, Kaufering, Germany). The sternal sensor is placed at the pressure point in the middle of the sternum. A piezo crystal is integrated, which is brought by the chest compression in sagittal motion. The RTF records the speed and time of each individual chest compression. From these two variables, it calculates chest compression depth in real time. Thoracic compression depth is recorded with an accuracy of ± 6.25 mm (15,16).

Ad 1.b. compression frequency:

The compression rate is subsequently measured via ECG artifacts from the monitor ECG. For this purpose, the ECG derivation II, by means of the self-adhesive paddles continuously measured and stored on a memory card (CF card). This ECG can be viewed and analyzed using the corView2 software. Chest compressions on the ECG can be distinguished from other electrical actions of the heart.

**ECG Interval with chest compression artifacts (corView2)**


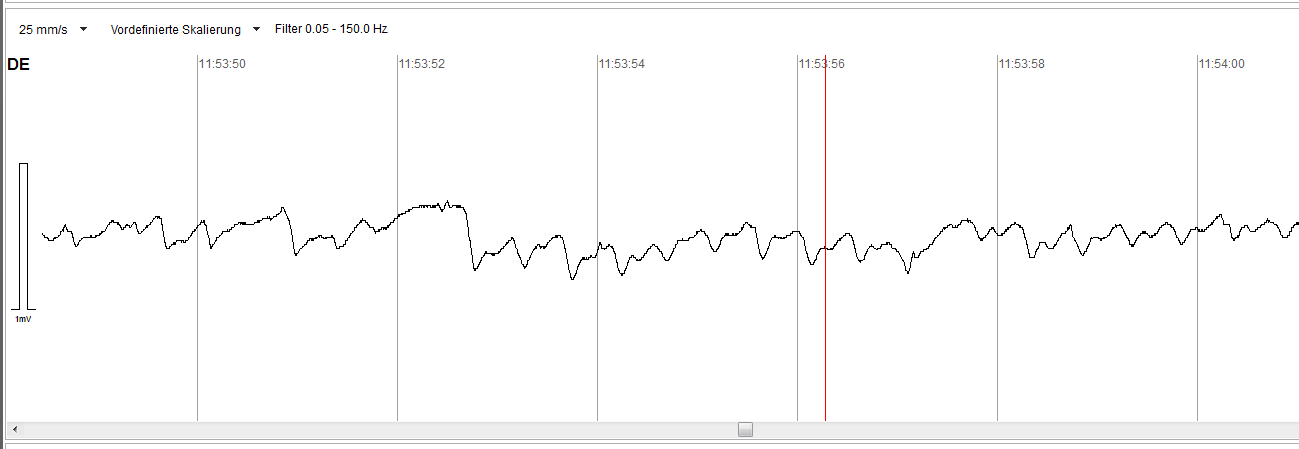


Ad 1.c.pauses in compression:

The interruptions of the compression ("hands-off") are subsequently analyzed via artefacts from the ECG.

- Total Breaks: All determined hands-off periods between the first and the last chest compression. A period without chest compression is defined with a threshold of at least 1.5 sec (hands-off periods are periods during which no chest compressions take place)
- Longest pause:

longest Hands-off pause in seconds

- Number of pauses >10s:

Number of all hands-off breaks greater than 10s

- Intermediate time interval until defibrillation ("Pre-Shock Pause"):

Average time in seconds of all pre-shock. Only calculated for hands-off periods with a delivered shock event. If there are multiple shocks within a hands-off period, the first defibrillation will always apply.

- Post-Shock pause:

Average time in seconds of all post-shock breaks. Only calculated for hands-off periods with a delivered shock event. If there are multiple shocks within a hands-off period, the first defibrillation will always apply.

- Peri-Shock pause:

Average time of all hands-off periods with one or more defibrillations.

- chest compression fraction:

Ratio of hands-on time to hands-off time (🡺 the ratio of times in chest compression is performed in relation to the times when no chest compressions is performed.

Ad 2. Short-time survival:

The resuscitation results (short-term survival) are recorded and evaluated in the German Resuscitation Registry®.

Indicators:

- Endpoint 1.1: Detecting a statistically significant change in the aforementioned qualities (chest compression frequency and interruption of compression) to phase 1 in phases 2 and 3. Detecting a change in thoracic compression depth in phases 2 and 3. Interruptions are defined as pauses greater than 1.5 s, in which at the same time the ECG has no indication of a perfusing heart rhythm (18).
- Endpoint 1.2 percent deviation from the ERC guidelines in terms of compression depth: 5.0-6.0 cm (19), compression frequency: 100 / min (19)
- Endpoint 2: Reaching hospital admission with spontaneous circulation (ROSC) documented in the resuscitation registry.

Inclusion criteria:

- All patients with a non-traumatic cardiac arrest who were 18 years old or older at the resuscitation attempt. Excluded are inmates.
- Resuscitations with chest compression time less than one minute are excluded

Design:

The study is build up in three phases:

In the first phase, 95 patient data are included, in phase 2 and 3 92 (total 279). This number of cases is taken from the biometric report. The city of Osnabrück and the district of Osnabrück have a combined population of 506,000 (as of Dec. 2013) (17). Every year about 300-400 out-of-hospital resuscitation attempts can be expected.

- **Phase 1:** Data is recorded without using the accelerometer (real-time feedback system). The analysis of chest compression frequency and interruptions is done via ECG evaluation. The data of the resuscitation register will be used to analyze the clinical data.
- **Phase 2:** Data is recorded using the feedback sensor without giving a real-time feedback to the Emergency medical staff. The analysis of chest compression frequency and interruptions is done via ECG evaluation. The data of the resuscitation register will be used to analyze the clinical data.
- **Phase 3**: Data is recorded using the feedback sensor and the Emergency medical staff receives a real-time feedback on chest compression quality. The analysis of chest compression frequency and interruptions is done via ECG evaluation. The data of the resuscitation register will be used to analyze the clinical data.

Data flow of the device data:

1. The patient data of each resuscitation are loaded via mobile data transfer to a server and is retrieved from there for evaluation. This corresponds to the usual procedure in the rescue services. The resuscitation missions are subsequently identified in the collective of missions.

2. The data for the resuscitation quality are loaded via mobile data transfer to a server and from there after identification of the inserts (see point 1) for evaluation. The data records are sent anonymously to the corpuls company:
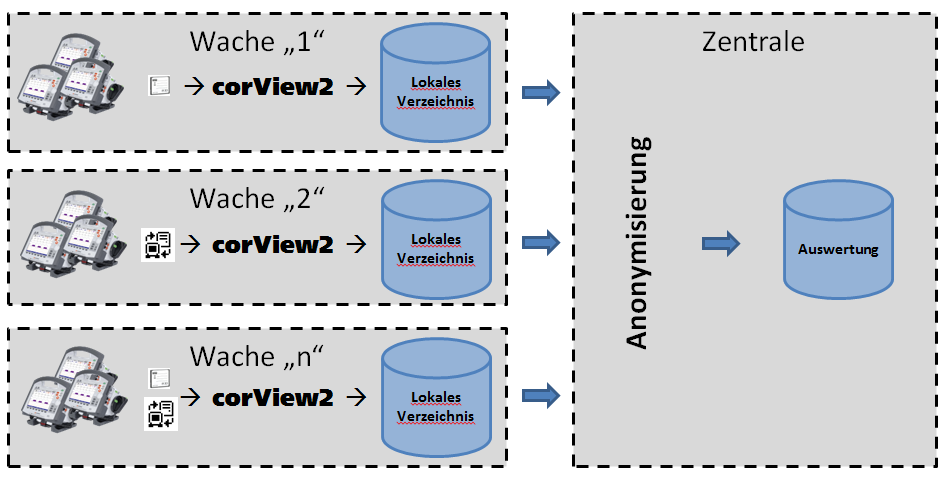


The anonymization will be done via filters. The individual data are listed below. Any data that could lead to patient identification, directly or in combination with other data, will be removed. The contents are no longer available in the evaluation database and can no longer be retrofitted.

| Patientendaten | | | | |
| --- | --- | --- | --- | --- |
| **Art der Daten** | identifizierend?  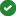 = ja 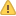 = evt. resp. in Kombination 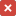 = nein | PHI?  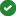 = ja 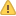 = evt. 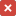 = nein | Maßnahmen | Ergebnis  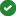 = k.P. 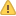 = Achtung 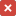 = Anw. BDSG |
| **Patienten-ID** | 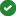 | 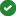 | Daten werden entfernt. | 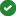 |
| **Fallnummer** | 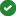 | 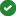 | Daten werden entfernt. | 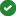 |
| **Vorname** | 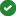 | 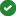 | Daten werden entfernt. | 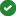 |
| **Nachname** | 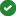 | 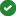 | Daten werden entfernt. | 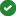 |
| **Geschlecht** | 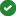 | 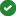 | Daten werden entfernt. | 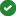 |
| **Geburtsdatum** | 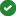 | 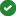 | Daten werden entfernt. | 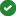 |
| **Alter** | 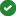 | 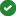 | Daten werden entfernt. | 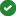 |
| **Gewicht** | 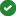 | 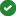 | Daten werden entfernt. | 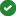 |
| **Größe** | 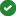 | 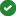 | Daten werden nicht erfasst | 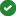 |
| **Adresse** | 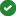 | 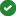 | Daten werden entfernt. | 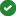 |
| **Ort** | 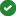 | 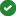 | Daten werden entfernt. | 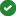 |
| **Versicherten-Nr** | 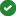 | 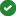 | Daten werden entfernt. | 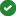 |
| **KK/Kostenträger** | 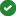 | 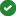 | Daten werden entfernt. | 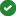 |
| **Versicherten Karten-Nr** | 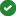 | 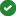 | Daten werden entfernt. | 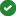 |
| **Transportmittel** | 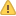 | 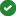 | Daten werden entfernt. | 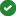 |
| **Funkkennung** | 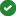 | 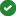 | Daten werden entfernt. | 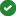 |
| **Geräte-ID** | 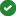  in Kombination mit Organisation |  | Daten werden entfernt. | 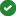 |
| **Zeitpunkt des Gerätestarts (Einsatzstart)** | 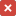 | 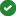 | identifizierende Daten entfernen, damit sind auch diese Daten kein PHI mehr | 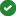 |
| **Dauer des Einsatzes (Einsatzende)** | 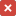 | 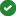 | identifizierende Daten entfernen, damit sind auch diese Daten kein PHI mehr | 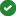 |
| **Seriennummer der einzelnen Gerätemodule** | 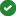 |  | Daten werden entfernt. | 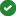 |
| **Seriennummer der einzelnen Geräteoptionen** | 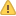 |  | Daten werden entfernt. | 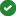 |
| **Kontinuierlich aufgezeichnete Kurven** | 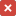 | 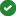 | identifizierende Daten entfernen, damit sind auch diese Daten kein PHI mehr | 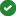 |
| **Kontinuierlich aufgezeichnete Vitalparameter** | 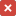 | 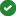 | identifizierende Daten entfernen, damit sind auch diese Daten kein PHI mehr | 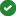 |
| **12-Kanal-EKG** | 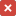 | 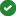 | identifizierende Daten entfernen, damit sind auch diese Daten kein PHI mehr | 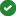 |
| **12-Kanal-EKG Vermessung, Diagnose und Therapieempfehlung (HES)** | 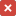 | 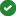 | identifizierende Daten entfernen, damit sind auch diese Daten kein PHI mehr | 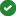 |

Data flow of patient data:

The mission is documented in the German Resuscitation Register and retrieved from there for evaluation. In the resuscitation register, the mission is pseudonymised stored under a mission number and can be retrieved from there for evaluation on the application number, without the data that allows a direct identification of the patient can be accessed.

Matching patient and device data:

The matching takes place on a computer of the University Hospital Münster as shown.

Statistics:

The statistical calculations were based on the knowledge from the study Kramer Johansen et al. "Quality of out-of-hospital cardiopulmonary resuscitation with real-time automated feedback: a prospective interventional study".

1. Endpoint 1.1: Phase 1 data are used as a reference for chest compression frequency and pauses in compression; Phase 2 data is used as a reference for the depth of compression. These phases are compared with the subsequent phases relating to quality.
2. Endpoint 1.2: The data is assessed phase-specifically for the percentage deviation from the ERC Reanimation Guidelines recommendations.
3. It is analysed whether phase-specific differences in short-term survival are recognizable.

Kompressionsfrequenz

Unterbrechung der Kompression

Kompressionstiefe

Phase 2

Phase 1

Phase 3

Phase 2

Phase 3

The multiple significance levels are set at 5%, and the power should always be ≥ 80% (error 2nd type ≤ 20%).

There are one or more comparisons for each target size. The multiple significance level of 5% is divided into Bonferronie targets in three parts (weighted):

1. For the comparisons concerning the depth of the compression, a local significance level of 1/4 * 5% is used. Here only the groups Phase 2 and Phase 3 are compared with each other with respect to the Mann-Whitney-U-test for the local significance level 1/4 * 5%.
2. For the comparisons regarding the compression frequency, a local significance level of 1/4 * 5% is used. Here the groups Phase1, Phase 2 and Phase 3 are compared with each other in pairs to the local significance level 1/4 * 5%. This results in 3 group comparisons. The multiple test problem is solved here by using the final test (for the pair comparisons the Mann-Whitney U test is used, for the 3 group comparisons (cutting hypothesis) the Kruskal-Wallis test is used).
3. For the comparisons concerning pauses in compression, a local significance level of 1/2 * 5% is used. Here, the groups Phase1, Phase 2 and Phase 3 are compared with each other in pairs to the local significance level 1/2 * 5%. This results in 3 group comparisons. The multiple test problem is solved here by using the final test (for the pair comparisons the Mann-Whitney U test is used, for the 3 group comparisons (cutting hypothesis) the Kruskal-Wallis test is used).

Powercalculation and number of cases:

The Phase 1 treatment group requires 95 patients, the Phase 2 treatment group requires 92 patients, and the Phase 3 treatment group requires 92 patients. In total, 95 + 92 + 92 = 279 patients are needed for this project.

1. For the comparisons of compression depth, with these numbers of patients a power of 82.1% is achieved.
2. For the comparisons of compression frequency, a power of 86.5% is achieved with these patient numbers (= probability that all three pair comparisons will yield a significant result).
3. For the comparisons of pauses in compression, with these patient numbers a power of 80.1% is achieved (= probability that all three pair comparisons yield a significant result).

Further planned statistical analyzes: In addition, quantitative estimates (Hodschges-Lehmann estimator) plus 95% confidence intervals for the group differences are calculated, and as an explorative question, all comparisons are additionally implemented separately for each gender.

Bibliography:

(1) Abella BS, Alvarado JP, Myklebust H, Edelson DP, Barry A, O'Hearn N, et al. (2005) Quality of cardiopulmonary resuscitation during in-hospital cardiac arrest. JAMA 293 (3): 305-310

(2) Abella BS, Sandbo N, Vassilatos P, Alvarado JP, O'Hearn N, Wigder HN, et al. (2005) Chest compression rates during cardiopulmonary resuscitation are suboptimal: a prospective study during in-hospital cardiac arrest. Circulation 111 (4): 428-434

(3) Christenson J, Andrusiek D, Everson-Stewart S, Kudenchuk P, Hostler D, Powell J, et al. (2009) Chest compression fraction determines survival in patients with out-of-hospital ventricular fibrillation. Circulation 120 (13): 1241-1247

(4) Kramer-Johansen J, Myklebust H, Wik L, Fellows B, Svensson L, Sorebo H, et al. (2006) Quality of out-of-hospital cardiopulmonary resuscitation with real time automated feedback: a prospective interventional study. Resuscitation 71 (3): 283-292

(5) Wik L, Kramer-Johansen J, Myklebust H, Sorebo H, Svensson L, Fellows B, et al. (2005) Quality of cardiopulmonary resuscitation during out-of-hospital cardiac arrest. JAMA 293 (3): 299-304

(6) Bjorshol CA, Myklebust H, Nilsen KL, Hoff T, Bjorkli C, Illguth E, et al. (2010) Effect of socioemotional stress on the quality of cardiopulmonary resuscitation during advanced life support in a randomized manikin study. Crit.Care Med.

(7) Goh J WD (2002) Aviat Space Environ Med 73 (73): 817–22

(8) Rittenberger JC, Guimond G, Platt TE, Hostler D (2006) Quality of BLS decreases with increasing resuscitation complexity. Resuscitation 68 (3): 365-369

(9) Lukas RP, Van Aken H, Engel P, Bohn A (2011) Real-time feedback systems for improvement of resuscitation quality. Anaesthesist

(10) Deakin CD, Nolan JP, Soar J, Sunde K, Koster RW, Smith GB, et al. (2010) European Resuscitation Council Guidelines for Resuscitation 2010 Section 4. Adult advanced life support. Resuscitation 81 (10): 1305-1352

(11) Koster RW, Baubin MA, Bossaert LL, Caballero A, Cassan P, Castren M, et al. (2010) European Resuscitation Council Guidelines for Resuscitation 2010 Section 2. Adult basic life support and use of automated external defibrillators. Resuscitation 81 (10): 1277-1292

(12) Edelson DP, Abella BS, Kramer-Johansen J, Wik L, Myklebust H, Barry AM, et al. (2006) Effects of compression depth and pre-shock pauses predict defibrillation failure during cardiac arrest. Resuscitation 71 (2): 137-145

(13) Berg RA, Sanders AB, Kern KB, Hilwig RW, Heidenreich JW, Porter ME, et al. (2001) Adverse hemodynamic effects of interrupting chest compressions for rescue breathing during cardiopulmonary resuscitation for ventricular fibrillation cardiac arrest. Circulation 104 (20): 2465-2470

(14) Eftestol T, Sunde K, Steen PA (2002) Effects of interrupting precordial compressions on the calculated probability of defibrillation success during out-of-hospital cardiac arrest. Circulation 105 (19): 2270-2273

(15) Bohn A, Van Aken H, Weber TP, Weber B, Lukas R (2012) Effects and limitations of an AED with audiovisual feedback for cardiopulmonary resuscitation. Resuscitation 83 (1): e9

(16) Zoll Medical Corporation [Erfinder]; Anonymous [Patentanmelder] (2011) Zoll E-Series (R) Benutzerhandbuch.

(17) [Landesbetrieb für Statistik und Kommunikationstechnologie Niedersachsen, 102 Bevölkerung – Basis Zensus 2011, Stand 31. Dezember 2013 (Tabelle K1020014)](http://www1.nls.niedersachsen.de/statistik/)

(18) Kramer-Johansen, J.; Edelson, D.P.; Losert, H.; et al Uniform reporting of measured quality of cardiopulmonary resuscitation. Resuscitation 2007; 412

(19) ERC Guidelines for Resuscitation; www.erc.edu
